# Supplementary material for: Histology Classification Highlights Differences in Efficacy of S-1 versus Capecitabine, in Combination with Cisplatin, for HER2-Negative Unresectable Advanced or Recurrent Gastric Cancer with Measurable Disease
Source: Cancers (Basel). 2022 Nov 18;14(22):5673. doi: 10.3390/cancers14225673 (PMC9688851; doi:10.3390/cancers14225673)
Supplement: Supplementary file 1 [file cancers-14-05673-s001.zip › cancers-2030216-supplementary.pdf]

Supplementary Table S1. Overall response for SP and XP in the integrated analysis

Arm	Number of cases	CR	PR	SD	PD	NE	ORR (95% CI)	DCR (95% CI)
SP	79	2	36	28	7	6	48.1% (36.7–59.6%)	83.5% (73.5–90.9%)
XP	83	1	41	18	18	5	50.6% (39.4–61.8%)	72.3% (61.4–81.2%)

Abbreviations not defined in text: NE, not evabuable.

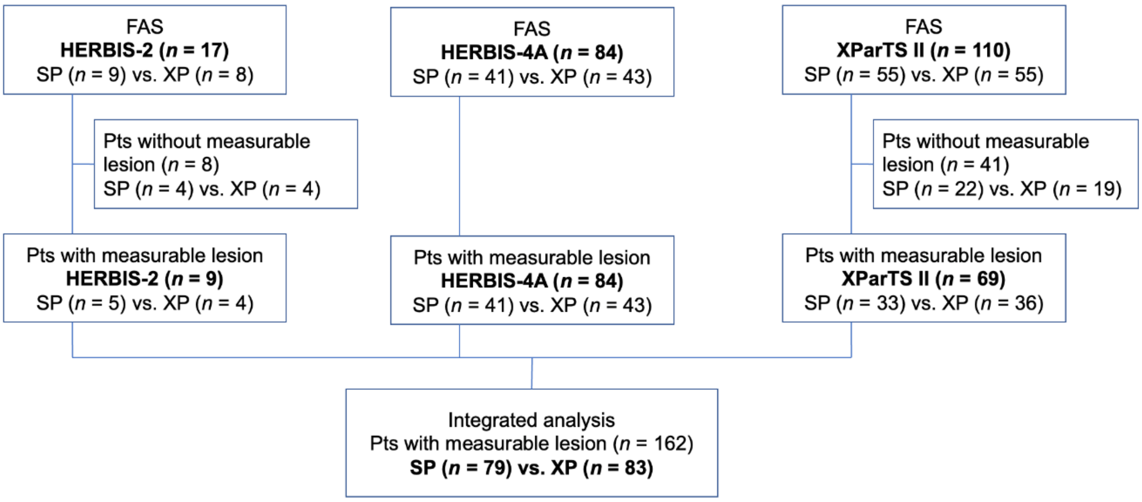

Supplementary Figure S1. Patient flow for the integrated analysis of the SP and XP arms. FAS, full analysis set; Pts, patients.

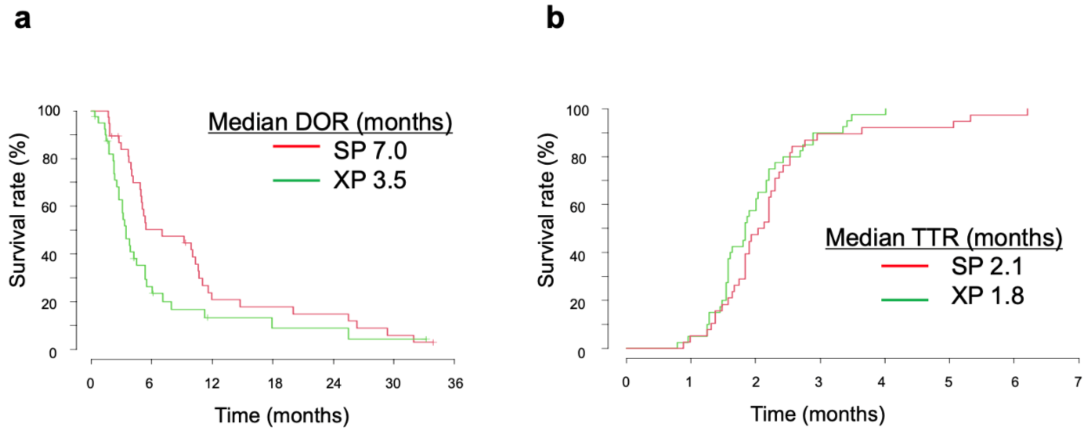

Supplementary Figure S2. Kaplan-Meier analysis of DOR (a) and TTR (b) for the SP and XP arms in the integrated analysis.

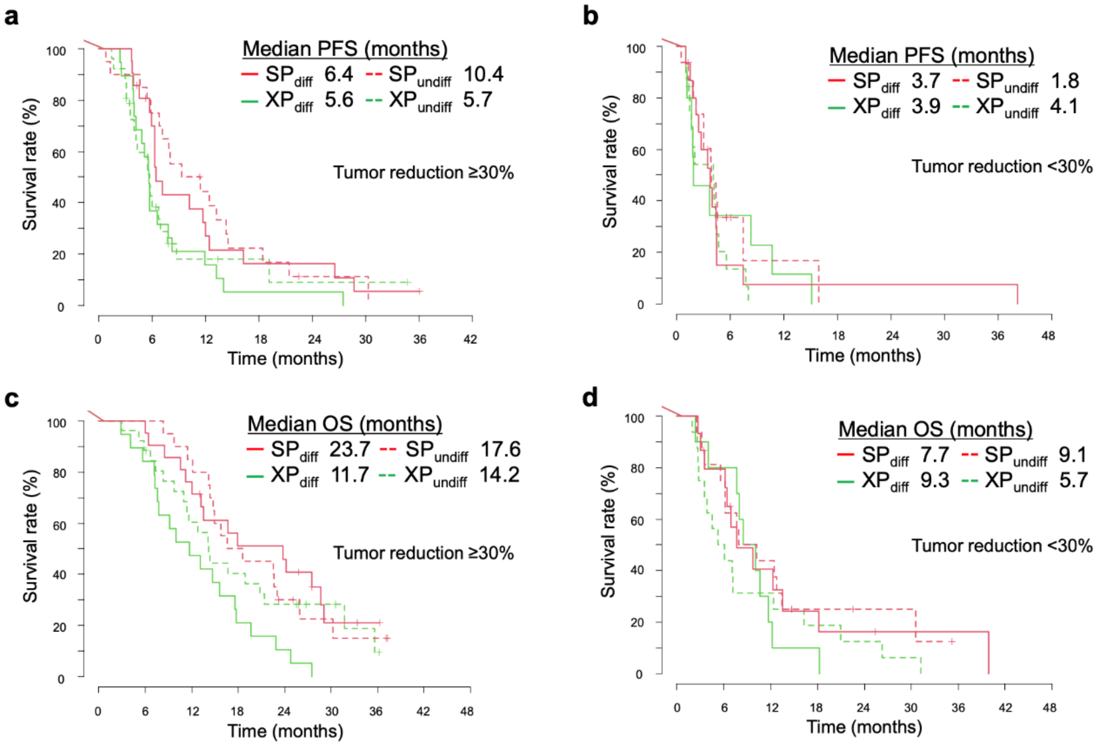

Supplementary Figure S3. Kaplan-Meier analysis of PFS (a and b) and OS (c and d) for patients with a tumor reduction of ≥30% (a and c) or <30% (b and d) from baseline according to differentiated (diff, solid lines) or undifferentiated (undiff, dotted lines) tumor types in the SP and XP arms of the integrated analysis.
